# Terahertz and infrared characteristic absorption spectra of aqueous glucose and fructose solutions

**DOI:** 10.1038/s41598-018-27310-7

**Published:** 2018-06-12

**Authors:** Chao Song, Wen-Hui Fan, Ling Ding, Xu Chen, Ze-You Chen, Kai Wang

**Affiliations:** 1 State Key Laboratory of Transient Optics and Photonics, Xi’an Institute of Optics and Precision Mechanics, Chinese Academy of Sciences, Xi’an, 710119 China; 2 University of Chinese Academy of Sciences, Beijing, 100049 China

## Abstract

In this paper, the terahertz (THz) and infrared (IR) characteristic absorption spectra of aqueous glucose solutions and aqueous fructose solutions with different concentrations were measured and studied. The absorption spectra of these two molecules in solid-state and in aqueous solutions were compared and analyzed, the significant effect of molecular adjacent environment on the molecular structure and vibrational mode was revealed. In addition, the THz and IR absorption spectra of these two isomers’ aqueous solutions were also compared and explored. No obvious differences were found from their IR absorption features measured at room temperature, while their THz absorption spectra do have the differences, indicating THz characteristic absorption spectra more suitable for the detection and identification of aqueous glucose and fructose solutions. The results are helpful to understand the influence of aqueous solutions environment on the molecular structures and vibrational modes of the materials, and also provide a theoretical reference for the quantum chemical calculation of biological macromolecules.

## Introduction

As the important biological molecules consisting of carbon (C) atom, hydrogen (H) atom and oxygen (O) atom, saccharides normally can be classified as four chemical groups: monosaccharides, disaccharides, oligosaccharides, and polysaccharides. In general, the monosaccharides and disaccharides are commonly referred to as sugars, which supply energy for the human body as the essential nutrient. In addition, saccharides also form the backbone of cells. For example, cellulose, hemicellulose and lignin are the main components of the plant cell wall, while peptidoglycan is the main component of the prokaryotic cell wall.

**Figure 1:**
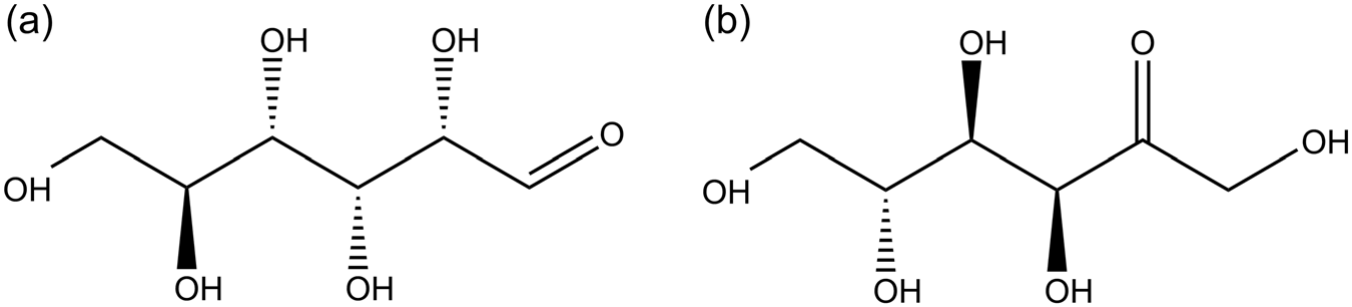
Molecular structures of (**a**) glucose and (**b**) fructose.

**Figure 2:**
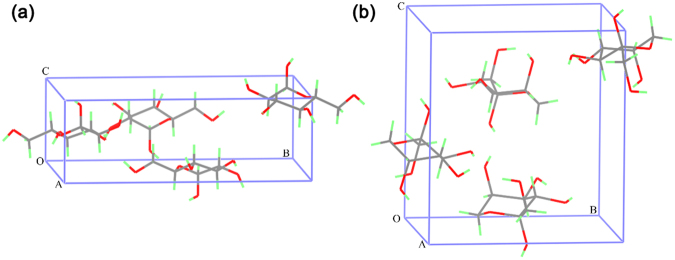
Molecular packing in unit cell of (**a**) glucose and (**b**) fructose.

Glucose and fructose are two kinds of monosaccharides required in human daily life. Both of them are white crystals at room temperature and have been used widely in the food industry. The pure aqueous solutions of both glucose and fructose are also transparent at room temperature, so it is very difficult to distinguish them from their appearance only. And also, as illustrated in Figs 1 and 2, glucose and fructose are isomers which have the same molecular formula, C_6_H_12_O_6_, but different arrangement of atoms involved. Crystal cell parameters of glucose^1^ are shown as follows: space group *P*2_1_2_1_2_1_ (*Z* = 4), *a* = 10.366 Å, *b* = 14.851 Å, *c* = 4.975 Å, *α*, *β*, *γ* = 90.0°. Crystal cell parameters of fructose^2^ are space group *P*2_1_2_1_2_1_ (*Z* = 4), *a* = 8.088 Å, *b* = 9.204 Å, *c* = 10.034 Å, *α*, *β*, *γ* = 90.0°. Clearly, there are four glucose molecules in the unit cell of glucose and four fructose molecules in the unit cell of fructose, respectively. And also, they have different symmetry, which affect the number of infrared (IR) vibrational mode and the characteristic absorption spectra in terahertz (THz) region.

So far, some previous work focused on THz or IR absorption spectra of glucose and fructose have been done^3–8^, but they are focused on either the experimental measurements of the solid-state matter only or just the theoretical calculations based on isolated molecule to analyze the origin of the characteristic absorption features. However, in the practical applications, the main functions of biomolecules are realized in aqueous solutions rather than in solid-state environment, although some researchers have achieved some advancements in characterization and quantification of glucose or fructose contents in fruits and biomedical applications^9–12^, the direct identification of aqueous glucose solutions and aqueous fructose solutions by using THz-TDS was studied much less due to the strong water absorption. Therefore, it is very meaningful to investigate the THz absorption spectra of aqueous glucose and fructose solutions as well as their origin and the effect of molecular adjacent environment on the molecular structure and vibrational mode.

It is well known that the vibrational absorption frequencies of organic intramolecular chemical bonds mainly locate in the IR band, while the absorption frequencies of intermolecular weak forces (such as hydrogen bonds and van der Waals’ force), molecular structure bending, dipole vibrations and rotational energy transitions as well as low frequency vibrations mainly locate in the THz band^13^. As the THz spectra is quite sensitive to the difference in organic molecular structure and their surrounding environment^4,14,15^, THz time-domain spectroscopy (THz-TDS) can be used as a new non-destructive detection technique to realize rapid and effective detection as well as qualitative identification of materials^16–20^. In addition, the THz-TDS based on coherent detection can not only measure the amplitude of the THz electric field directly, but also obtain the phase of the THz electric field simultaneously. Therefore, the signal-to-noise ratio of THz-TDS is much higher than that of Fourier transform IR spectroscopy (FTIR), which is non-coherent detection^21^. However, although FTIR can obtain amplitude information only, it can cover the very broad range from far-IR to visible, so it is more suitable to measure the absorption spectrum over mid-IR band where THz-TDS cannot be utilized efficiently. Thus, the combination of FTIR and THz-TDS technology is valuable in studying the characteristic absorption spectra of materials, which can effectively achieve technical complementarities and improve the characteristic absorption spectra information on the corresponding materials.

In this paper, the THz and IR absorption spectra of aqueous glucose solutions and aqueous fructose solutions with different concentrations has been measured at room temperature. By comparing the characteristic absorption spectra of glucose and fructose aqueous solutions in different concentrations, the influence of the surrounding environment on their molecular structures and vibration modes was explored. By analyzing the THz and IR characteristic absorption spectra of solid-state glucose and fructose as well as their corresponding aqueous solutions, the physical mechanism that the characteristic absorption features of glucose and fructose molecules broadened in their aqueous solutions was revealed. In addition, we also found that IR absorption spectra of these two isomers’ solutions were almost same, but the THz absorption spectra were a little different. Therefore, THz spectroscopy can be more promising in identifying aqueous glucose solutions and aqueous fructose solutions.

## Results and Discussion

### Terahertz absorption spectra of aqueous glucose solutions and aqueous fructose solutions

**Figure 3:**
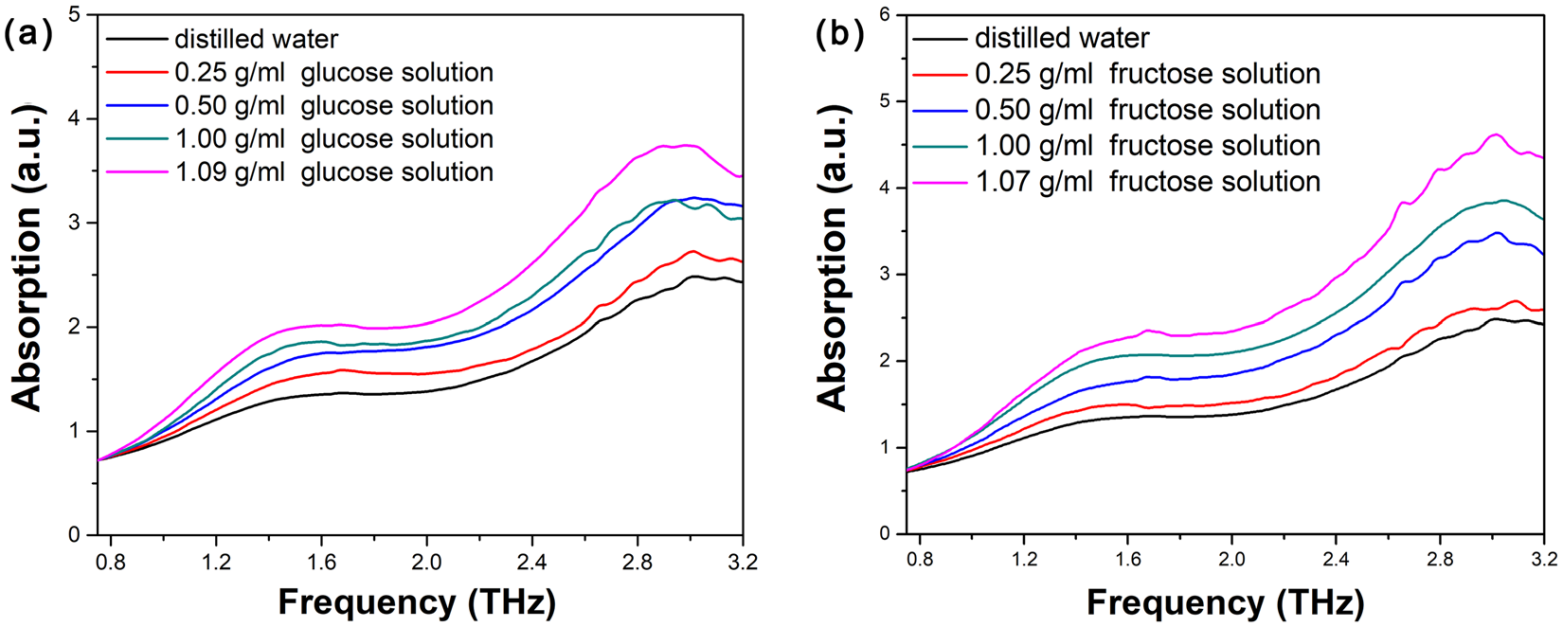
THz absorption spectra of (**a**) distilled water and different concentrations of aqueous glucose solutions, (**b**) distilled water and different concentrations of aqueous fructose solutions.

The THz absorption spectra of aqueous glucose solutions and aqueous fructose solutions with different concentrations as well as distilled water were measured at room temperature, as shown in Fig. 3. Compared with distilled water, the absorption of both aqueous glucose solutions and aqueous fructose solutions is stronger, and also the absorption intensities increase with the concentrations increasing, indicating clearly the THz absorption spectra of aqueous glucose solutions and aqueous fructose solutions affected by the amount of glucose or fructose molecules involved.

**Figure 4:**
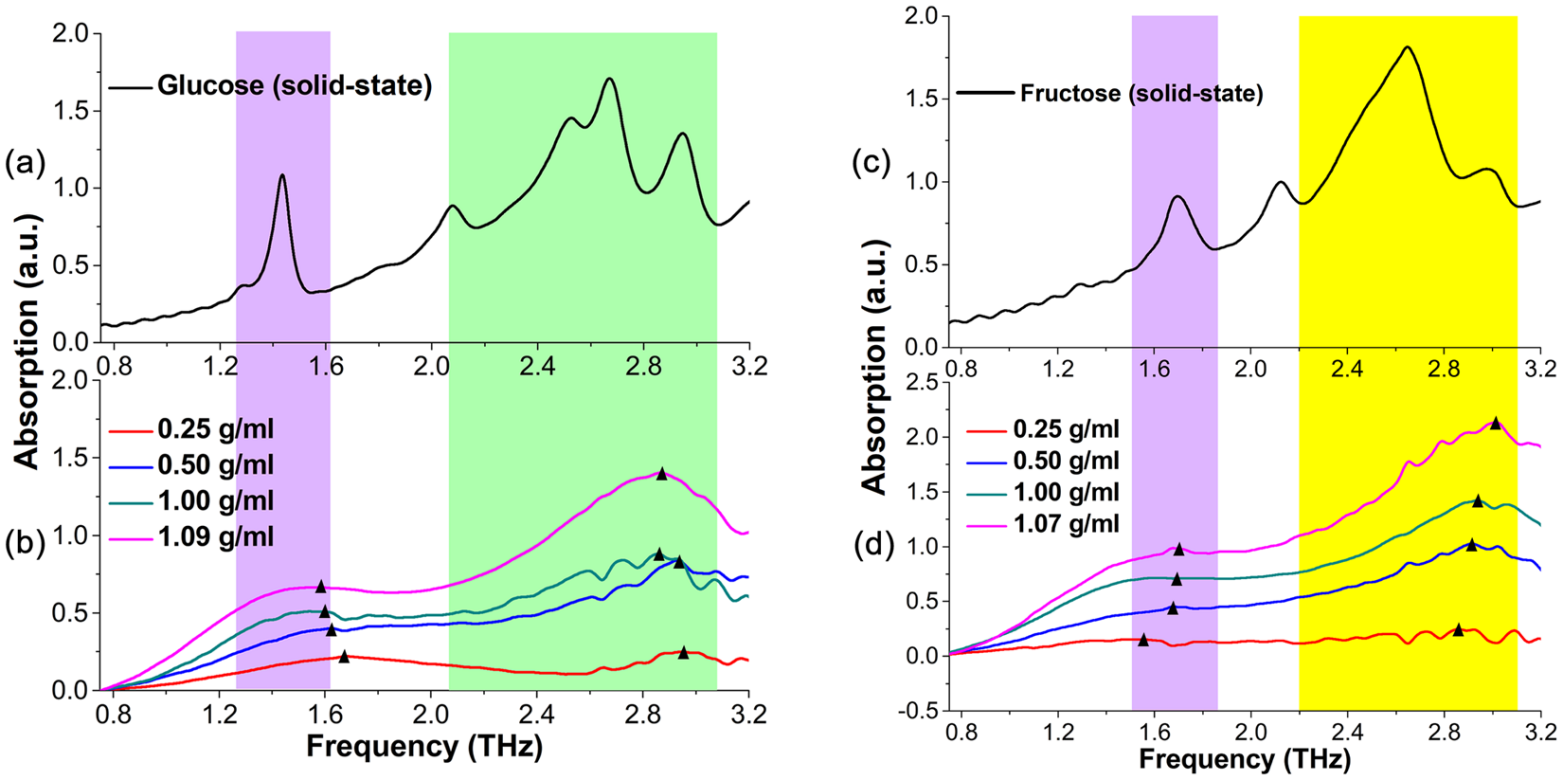
THz absorption spectra of (**a**) solid-state glucose, (**b**) pure glucose molecules in aqueous solutions with different concentrations, (**c**) solid-state fructose, and (**d**) pure fructose molecules in aqueous solutions with different concentrations.

In addition, because distilled water also shows strong absorption in the THz range, the THz absorption spectra presented in Fig. 3 are actually the combined THz absorption spectra of distilled water and aqueous glucose solutions or aqueous fructose solutions. After the contribution of distilled water is deducted, the pure THz absorption spectra of glucose or fructose molecules in aqueous solutions are obtained, as shown in Fig. 4(b) and (d), respectively. As the direct comparison, the THz absorption spectra of glucose or fructose molecules in solid-state have also been measured, as presented in Fig. 4(a) and (c).

As what can be observed in Fig. 4(b), the absorbance of 0.25 g/ml aqueous glucose solution is quite small, and the absorbance of aqueous glucose solutions go up gradually with the concentrations increasing until the aqueous glucose solutions gets saturated at concentration of 1.09 g/ml, where two consistent broad absorption bands around 1.58 THz and 2.87 THz can be observed clearly. According to the THz absorption spectra of solid-state glucose in Fig. 4(a), it can be seen that solid-state glucose molecules have strong characteristic absorption at 1.42 THz, 1.78 THz, 2.05 THz, 2.51 THz, 2.64 THz and 2.91 THz, and the first three peaks are very likely to make contribution at certain extent to the broad absorption of aqueous glucose solutions centered around 1.58 THz at concentration of 1.09 g/ml, and the last three peaks are likely to contribute to the broad absorption of aqueous glucose solutions centered on 2.87 THz. To make these more clearly, the triangle marks labeled in Fig. 4(b) represent the THz absorption peaks of the aqueous glucose solutions in different concentrations. It is clearly observed that the absorption peaks of aqueous glucose solutions in different concentrations shift separately to the characteristic absorption peaks of solid-state glucose at 1.42 THz and 2.91 THz as the proportions of glucose molecules rise up gradually in the aqueous solutions, especially in the case of the saturated aqueous glucose solutions at concentration of 1.09 g/ml.

As for the THz absorption spectra of aqueous fructose solutions illustrated in Fig. 4(d), the situation is roughly similar as the THz absorption spectra of aqueous glucose solutions shown in Fig. 4(b). The absorbance of 0.25 g/ml aqueous fructose solution is nearly zero except for several very small fluctuations from 0.75 THz to 3.2 THz and the absorbance of aqueous fructose solutions rise up gradually with the concentrations increasing until the aqueous fructose solutions gets saturated at concentration of 1.07 g/ml, where two consistent broad absorption bands around 1.68 THz and 3.02 THz can be clearly observed. On the other hand, strong characteristic absorption peaks of solid-state fructose at 1.69 THz, 2.12 THz, 2.43 THz, 2.65 THz and 2.95 THz can be seen in Fig. 4(c). Therefore, the broad absorption band of aqueous fructose solutions centered on 1.68 THz could be mainly originated from the strong characteristic absorption peak of solid-state fructose at 1.69 THz related to the translational motion of fructose molecule, and the other broad absorption band of aqueous fructose solutions centered on 3.02 THz may mainly originate from the strong characteristic absorption peak of solid-state fructose at 2.95 THz, which is relevant to the twisting of CH_2_OH functional group (see also Table S2 in Supplementary Information). To make these more clearly, the triangle marks labeled in Fig. 4(d) represent the THz absorption peaks of the aqueous fructose solutions in different concentrations. It can be clearly observed that the absorption peaks of aqueous fructose solutions in different concentrations move separately towards to the characteristic absorption peaks of solid-state fructose at 1.69 THz and 2.95 THz gradually with the increasing proportions of fructose molecules in the aqueous solutions, especially in the case of the saturated aqueous fructose solution at concentration of 1.07 g/ml.

Very interestingly, strong characteristic absorption peaks of solid-state fructose at 2.12 THz, 2.43 THz and 2.65 THz shown in Fig. 4(c) are quite difficult to be observed in the THz absorption spectra of aqueous fructose solutions illustrated in Fig. 4(d), especially the strong characteristic absorption peak of solid-state fructose at 2.12 THz shown clearly in Fig. 4(c), indicating the strong influence of aqueous solutions environment on the molecular structure and vibrational modes of the material. The similar situation can also be observed from solid-state glucose and aqueous glucose solutions, as shown in Fig. 4(a) and (b).

Therefore, as mentioned above, the THz absorption intensity of aqueous glucose or fructose solutions increase with the concentrations increasing, and the characteristic absorption spectra of glucose or fructose molecules in aqueous solutions are broadened compared to the characteristic absorption spectra measured in solid-state. Moreover, a broad absorption band in aqueous solutions may consist of several adjacent characteristic absorption peaks appeared in solid-state, indicating the glucose or fructose molecules affected strongly by the water molecules in aqueous solutions, the variation of their intramolecular and intermolecular interactions also leads to the significant difference between the absorption spectra in aqueous solutions and the absorption spectra in solid-state.

### Infrared absorption spectra of aqueous glucose solutions and aqueous fructose solutions

**Figure 5:**
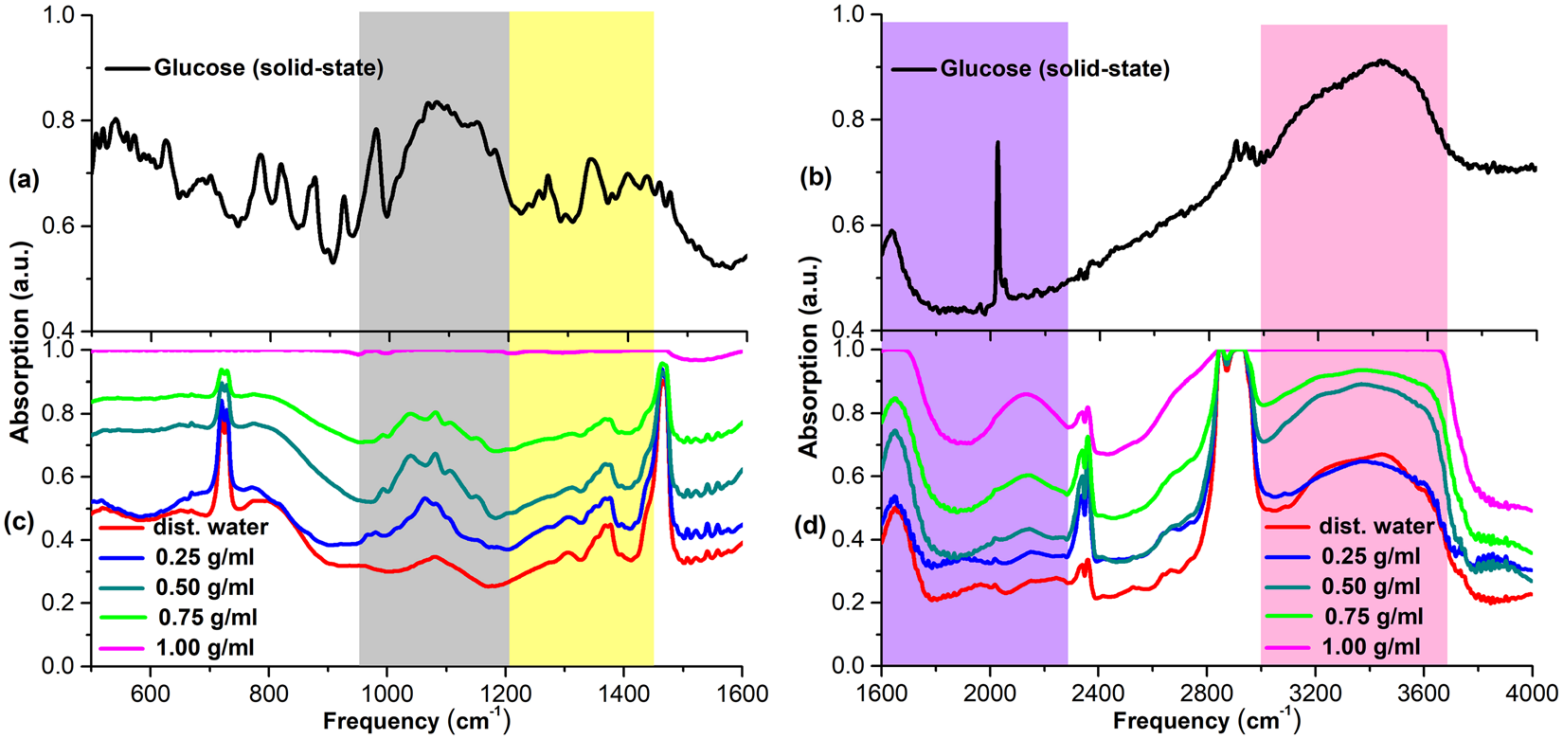
Infrared absorption spectra of solid-state glucose in different frequency ranges (**a**) 500–1600 cm^−1^ (**b**) 1600–4000 cm^−1^; Infrared absorption spectra of distilled water and different concentrations of aqueous glucose solutions in different frequency ranges (**c**) 500–1600 cm^−1^ (**d**) 1600–4000 cm^−1^.

Figure 5 presents the IR absorption spectra of solid-state glucose and aqueous glucose solutions with different concentrations, measured in the range of 500–4000 cm^−1^. It can be seen in Fig. 5(c) that there are four narrow absorption bands centered 993.3 cm^−1^, 1039.6 cm^−1^, 1107.1 cm^−1^ and 1149.5 cm^−1^ in the absorption spectra of aqueous glucose solutions, which are not appeared in the absorption spectra of distilled water in the range of 950–1200 cm^−1^. Compared the IR absorption spectra of solid-state glucose to that of aqueous glucose solutions, four absorption peaks of aqueous glucose solutions correspond to the absorption features of the solid-state glucose molecules at 977.9 cm^−1^, 1080.1 cm^−1^, 1147.6 cm^−1^ and 1176.5 cm^−1^, respectively, which originate from intramolecular vibrations, such as CH_2_OH group deformation vibration, carbon ring deformation vibration, C-H bond stretching vibration and C-H bond wagging vibration (see also Table S3 in Supplementary Information). In the 1200–1430 cm^−1^ band, both the aqueous glucose solutions and distilled water has a broad absorption band centered at 1271.0 cm^−1^ and 1377.1 cm^−1^, and the absorption of aqueous glucose solutions is much stronger than that of distilled water, indicating both water molecules and glucose molecules in the solution making contribution to the absorption bands. In addition, these characteristic absorption bands are consistent with the absorption band of solid-state glucose molecules at 1265.2 cm^−1^ and 1338.5 cm^−1^. In the 1600–2300 cm^−1^ band, as illustrated in Fig. 5(d), the absorption of aqueous glucose solutions at 1652.9 cm^−1^ and 2137.0 cm^−1^ increase with the concentrations increasing, and the characteristic absorption at 1652.9 cm^−1^ is consistent with the absorption of solid-state glucose at 1624.0 cm^−1^. However, due to the influence of distilled water molecules on glucose molecules, the narrow absorption band at 2030.0 cm^−1^ was broadened as a broad absorption band centered at 2137.0 cm^−1^. Moreover, it can be seen that the broadband absorption of aqueous glucose solutions in the range of 3000–3700 cm^−1^ mainly originate from strong absorption of glucose molecules and water molecules.

**Figure 6:**
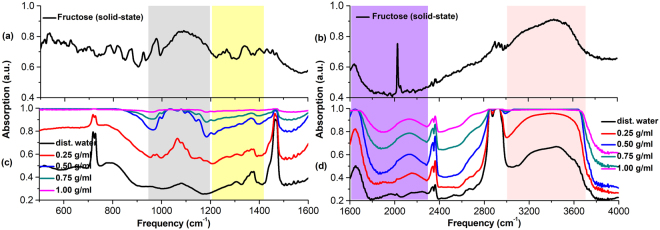
Infrared absorption spectra of solid-state fructose in different frequency ranges (**a**) 500–1600 cm^−1^ (**b**) 1600–4000 cm^−1^; Infrared absorption spectra of distilled water and different concentrations of aqueous fructose solutions in different frequency ranges (**c**) 500–1600 cm^−1^ (**d**) 1600–4000 cm^−1^.

Figure 6 illustrates the IR absorption spectra of solid-state fructose and aqueous fructose solutions with different concentrations. It can be observed in Fig. 6(c) that the absorption spectra of the aqueous fructose solutions in the range of 950–1200 cm^−1^ has an absorption peak around 979.8 cm^−1^ and a broad absorption band near 1064.7 cm^−1^, which correspond to the absorption peak of solid-state fructose at 975.9 cm^−1^ and the broad absorption band near 1089.7 cm^−1^ originated from multiple intramolecular vibrations, respectively (see also Table S4 in Supplementary Information); in the frequency range of 1200–1430 cm^−1^, both distilled water and aqueous fructose solutions present three broad absorption bands around 1269.1 cm^−1^, 1307.7 cm^−1^ and 1367.5 cm^−1^, and also the absorption of aqueous fructose solutions is stronger than that of distilled water, indicating these three characteristic absorption of aqueous fructose solutions attributed to the absorption of fructose molecules and distilled water molecules together. Moreover, Fig. 6(a) also shows that the solid-state fructose molecules present characteristic absorption at 1265.2 cm^−1^, 1338.5 cm^−1^ and 1400.3 cm^−1^. In the range of 1600–2300 cm^−1^ shown in Fig. 6(d), the characteristic absorption of aqueous fructose solutions at 1647.1 cm^−1^ and 2127.4 cm^−1^ increase with the concentrations increasing, and the absorption of aqueous fructose solutions at 1647.1 cm^−1^ is consistent with the characteristics absorption peak of solid-state fructose at 1637.5 cm^−1^. Considering the influence of aqueous solutions environment on fructose molecules, the interaction force between fructose molecules will be weakened in aqueous fructose solutions compared to that in solid-state fructose, as a result, the broadband absorption at 2127.4 cm^−1^ could be possibly attributed to the broad absorption of narrow absorption band at 2025.1 cm^−1^. In the range of 3000–3700 cm^−1^, the broad absorption band of aqueous fructose solutions can also be attributed to the absorption band of solid-state fructose at the corresponding frequency.

### Comparison between the characteristic absorption spectra of aqueous glucose and fructose solutions in terahertz and infrared region

**Figure 7:**
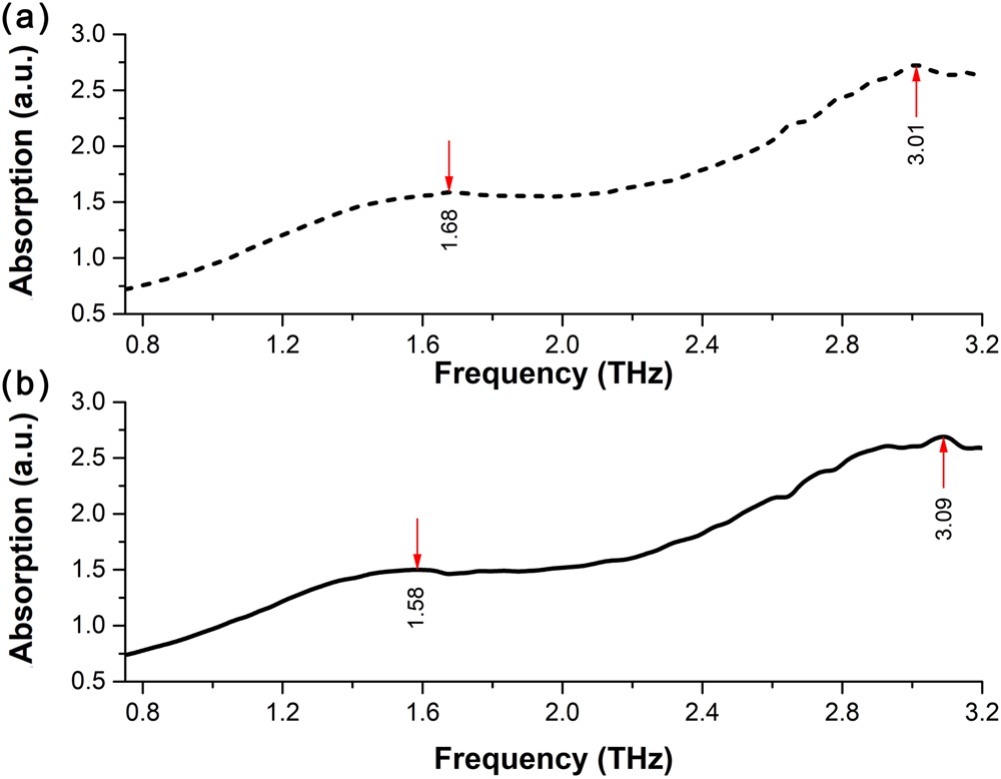
Terahertz absorption spectra of 0.25 g/ml aqueous solutions of (**a**) glucose and (**b**) fructose.

**Figure 8:**
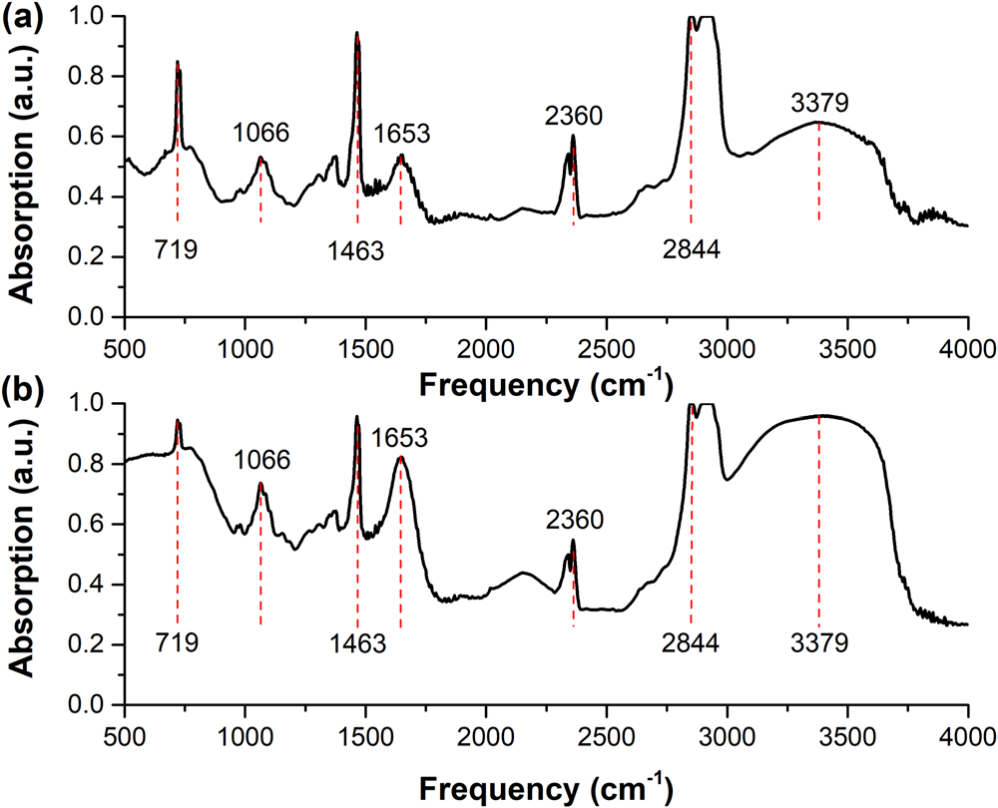
Infrared absorption spectra of 0.25 g/ml aqueous solutions of (**a**) glucose and (**b**) fructose.

Because the IR absorption spectra of aqueous glucose solutions and aqueous fructose solutions get saturated over concentration of 0.25 g/ml, the direct comparison in THz and IR region have to be made between the characteristic absorption spectra of aqueous glucose and fructose solutions at concentration of 0.25 g/ml without the contribution of distilled water deducted, as shown in Figs 7 and 8, respectively.

It can be seen in Fig. 8 that there is no significant difference of the IR characteristic absorption spectra of these two isomers in aqueous solution at the concentration of 0.25 g/ml in the IR range (500–4000 cm^−1^), which could be very difficult to distinguish aqueous glucose solution and aqueous fructose solution via the IR absorption spectra. The phenomenon mainly originates from the fact that intramolecular interactions contribute to the IR characteristic absorption of glucose and fructose, and the molecular structures of these two isomers are quite similar. However, as shown in Fig. 7, the spectral absorption peaks of these two isomers even in aqueous solutions at the concentration of 0.25 g/ml show the difference in the THz range. Considering the significant THz spectral differences between solid-state glucose in Fig. 4(a) and fructose in Fig. 4(c), it could be interpreted by their different intermolecular interactions^4^, such as the molecule arrangement and crystal packing shown in Fig. 2. Although the intermolecular interactions of glucose molecules or fructose molecules in aqueous solutions become weaker compared to that of glucose molecules or fructose molecules in solid-state, some of THz characteristic absorption features of these two isomers still exist, so THz absorption spectra can be utilized in identifying aqueous glucose solutions and aqueous fructose solutions.

## Conclusion

In summary, THz and IR absorption spectra of solid-state glucose and fructose as well as their corresponding aqueous solutions have been studied. By comparing the THz and IR absorption of aqueous glucose or fructose solutions with different concentrations, it can be observed that the absorption intensities increase with the concentrations increasing. This phenomenon verifies that both distilled water molecules and glucose or fructose molecules have made contribution to the measured absorption spectra. By comparing the THz and IR absorption of aqueous glucose or fructose solutions with their corresponding absorption features in solid-state, it is obvious that the characteristic absorption features of aqueous glucose or fructose solutions become broadened, indicating the intramolecular and intermolecular interactions of the glucose and fructose molecules weakened due to the influence of distilled water molecules. Finally, we also made a comparison between the THz and IR absorption spectra of aqueous glucose and fructose solutions. The results show that the THz spectroscopy is more suitable than the IR spectroscopy for the identification of glucose and fructose, whatever in solid-state or in aqueous solutions. This is mainly because glucose and fructose are isomers and their molecular structures are similar, and the IR resonance absorption mainly originates from intramolecular vibrations. However, their molecular arrangement and intermolecular interactions are different where most of the THz resonance absorption originates. Although the solvent (water) in solution weakens the interaction force between the solute (glucose or fructose) molecules, the differences between their absorption spectra in the THz range still exist. These results fully reveal the significant influence of the surrounding environment on the molecular structures and its vibration modes, and provide a promising method of the rapid and effective identification of matter. And also, the results lay a solid foundation for the application of THz and IR spectroscopy in the study of the structures and properties of organic materials.

## Methods

### Sample preparation

Glucose (CAS-number 50-99-7) and fructose (CAS-number 57-48-7) were purchased from Chengdu Chemical Reagent Factory. The two types of samples were in analytical grade purity (>99%) and directly used for measurements without further purification. As for the preparation of solid-state samples, in order to reduce particle scattering, the pure samples purchased were firstly grinded into fine powder by pestle and agate mortar. To reduce the intense absorption of the samples measured, they have to be diluted with high-density polyethylene (HDPE) for the measurement in the THz range or potassium bromide (KBr) for the measurement in the IR range. Finally, the samples were pressed into surface-smooth pellets under certain pressure. Besides, all the measurements were carried out in dry air conditions (relative humidity <1%) to minimize the absorption of water vapor.

On the other hand, to measure the absorption spectra of aqueous glucose and fructose solutions, the distilled water was prepared as the solvent and the pure samples were grinded into fine powder by pestle and agate mortar at first. Then, the powder was dissolved into the distilled water in different proportions. After that, the aqueous glucose or fructose solutions with different concentrations were successively injected into the particular liquid sample cell and the inlet was sealed. Finally, the liquid sample cell was placed in the focal plane of the beam path and the spectrum was measured when the relative humidity in the closed test environment dropped below 1%.

### Experimental apparatus

The THz absorption spectra of samples were measured by using a TPS-3000 spectrometer (TeraView Ltd., UK)^22^ in the range of 0.1–4.0 THz with frequency resolution of 0.03 THz. The IR absorption spectra of samples were measured by using a VERTEX 70 spectrometer (Bruker Optics Inc., GER) in the range of 500–4000 cm^−1^ (15–120 THz) with frequency resolution of 2 cm^−1^ (0.06 THz).

### Data availability statement

The datasets generated during and/or analyzed during the current study are available from the corresponding author on reasonable request.

## Electronic supplementary material

Supplementary Information
