# Supplementary material for: Terahertz and infrared characteristic absorption spectra of aqueous glucose and fructose solutions
Source: Sci Rep. 2018 Jun 12;8:8964. doi: 10.1038/s41598-018-27310-7 (PMC5997655; doi:10.1038/s41598-018-27310-7)
Supplement: Supplementary file 1 — Supplementary Information [file 41598_2018_27310_MOESM1_ESM.pdf]

Supplementary Information for
Terahertz and infrared characteristic absorption spectra of aqueous glucose and fructose solutions

Chao Song[1, 2], *Wen-Hui Fan*[1, *], *Ling Ding*[1,2], *Xu Chen*[1,2], *Ze-You Chen*[1, 2] *and Kai Wang* [1, 2]

[1] State Key Laboratory of Transient Optics and Photonics, Xi'an Institute of Optics and Precision Mechanics, Chinese Academy of Sciences, Xi'an 710119, China

[2] University of Chinese Academy of Sciences, Beijing 100049, China

[*] To whom correspondence should be addressed. [*]fanwh@opt.ac.cn

To qualitatively clarify the effect of surrounding environment on the molecular structure and vibrational mode, the relevant theoretical calculation based on isolated-molecules and unit cells of solid-state glucose and fructose were also carried out at the meantime of our experimental measurements of glucose and fructose based on THz-TDS and FTIR. The calculations based on isolated-molecules utilized linear combination theory of atomic orbitals with Moller-Plesset perturbation theory (MP2)[1] and Becke-Three-Lee-Yang-Parr (B3LYP) functional[2] with the 6-311+G (d, p) Gaussian basis set, respectively. The calculations based on unit cell used the plane-wave density functional theory (DFT) with generalized gradient approximation (GGA)[3]. Specifically, the Perdew-Burke-Ernzerhof (PBE)[4] and Perdew-Wang (PW91)[5] exchange correlation functional were used in the calculations based on unit cell. Norm-conserving Kleinman-Bylander pseudopotentials[6] were utilized to describe electron-ion interactions. The plane-wave cutoff energy was set to 1200 eV; the total energy was converged to 10^{-8} eV/atom; the maximum forces between atoms were less than 10^{-5} eV/Å. Moreover, all the optimization of the crystal structures was conducted within the constraints of the unit cell. Compared with the experimental measurement of absorption features of solid-state glucose and fructose, Table S1, Table S2, Table S3 and Table S4 give a clear explanation about the origin of corresponding absorption peaks.

Table S1. The assignment of the THz absorption features and mode description of solid-state glucose

Experiment (THz)	Theoretical calculation of vibration modes (THz)				
	MP2 （isolated-molecule）	B3LYP （isolated-molecule）	PBE （unit cell）	PW91 （unit cell）	Mode description
1.27	-	-	1.26	-	Translational motion of molecules along *a*-axis
1.42	-	-	1.50	1.37	Rotation of molecules around *a*-axis
1.78	-	-	1.75	1.70	Rotation of molecules around *c*-axis
2.05	1.98	1.93	2.03	2.10	Twisting of -CH_2OH
-	-	-	2.18	2.21	Rotation of molecules around *b*-axis
2.51	-	-	2.38	2.35	Translational motion of molecules along *b*-axis
-	-	-	2.46	-	Rotation of molecules around *c*-axis
2.64	2.90	2.68	2.47	2.43	Twisting of -CH_2OH
2.91	3.23	3.09	2.70	2.75	Twisting of -CH_2OH
-	-	-	2.87	2.95	Translational motion of molecules along *c*-axis
-	-	-	3.10	-	Rotation of molecules around *c*-axis
-	-	-	3.15	-	Translational motion of molecules along *a*-axis
3.32	-	-	3.35	3.10	Rotation of molecules around *a*-axis
3.48	-	-	3.69	3.49	Rotation of molecules around *b*-axis
3.74	-	-	3.88	3.60	Rotation of molecules around *a*-axis

Table S2. The assignment of the THz absorption features and mode description of solid-state fructose

Experiment (THz)	Theoretical calculation of vibration modes（THz）				
	MP2 （isolated-molecule）	B3LYP （isolated-molecule）	PBE （unit cell）	PW91 （unit cell）	Mode description
1.69	-	-	1.74	1.71	Translational motion of molecules along *a*- axis
-	-	-	1.90	-	Translational motion of molecules along *c*- axis
2.12	2.30	2.27	2.10	2.08	Deformation of carbon ring
2.43	-	-	2.29	2.26	Rotation of molecules around *b*-axis
			2.32	2.31	Rotation of molecules around *b*-axis
2.65	-	-	2.53	2.46	Rotation of molecules around *c*-axis
-	-	-	2.64	2.53	Rotation of molecules around *c*-axis
2.95	2.98	2.98	2.97	3.18	Twisting of $-CH_2OH$
-	-	-	3.01	3.24	Translational motion of molecules along *b*- axis
3.24	-	-	3.28	3.55	Rotation of molecules around *b*-axis
3.56	3.64	3.65	3.56	3.85	Twisting of $-CH_2OH$
3.68	-	-	3.64	3.89	Rotation of molecules around *c*-axis
3.84	-	-	3.72	-	Rotation of molecules around *b*-axis

Table S3. The assignment of the IR absorption features and mode description of solid-state glucose

Experiment（cm^{-1}）	Theoretical calculation of vibration modes（cm^{-1}）				
	MP2（isolated-molecule）	B3LYP（isolated-molecule）	PBE（unit cell）	PW91（unit cell）	Mode description
628.8	604.4	588.8	618.6	610.6	Wagging of O-H
686.6	645.8	626.4	691.6	-	Wagging of O-H
783.1	781.4	-	743.9	702.9	Wagging of O-H
817.8	-	-	797.2	751.2	Deformation of carbon ring
875.6	874.2	-	829.6	843.2	Wagging of O-H
923.9	918.0	917.4	1010.6	1032.6	Stretching of C-C
977.9	1028.4	1009.6	1061.6	1074.6	Deformation of -CH_2OH
1080.1	1099.0	1065.8	1103.2	1093.6	Deformation of carbon ring
1147.6	1142.6	1094.4	1133.9	1120.2	Stretching of C-H
1176.5	1179.2	1149.2	1171.9	1166.6	Wagging of C-H
1265.2	1268.6	1236.4	1237.6	1227.9	Wagging of C-H
1338.5	1329.4	1330.0	1348.6	1364.2	Wagging of C-H
1624.0	-	-	-	-	-
2030.0	-	-	-	-	-
2898.9	3050.2	2956.0	2873.9	2811.6	Stretching of C-H
2933.6	3111.8	3006.8	2919.9	2871.6	Symmetrical stretching of H-C-H
3200.0	-	-	3153.9	3040.9	Stretching of O-H
-	-	-	3176.6	3073.6	Stretching of O-H
3450.0	-	-	3265.2	3212.6	Stretching of O-H
-	-	-	3298.6	3327.9	Stretching of O-H
-	-	-	3331.6	3446.9	Stretching of O-H

Table S4. The assignment of the IR absorption features and mode description of solid-state fructose

Experiment （cm^{-1}）	Theoretical calculation of vibration modes（cm^{-1}）				
	MP2 （isolated-molecule）	B3LYP （isolated-molecule）	PBE （unit cell）	PW91 （unit cell）	Mode description
538.1	514.0	521.0	514.1	513.9	Wagging of O-H
623.0	630.2	633.4	628.9	628.1	Wagging of O-H
694.3	-	-	662.5	663.0	Wagging of O-H
786.9	783.2	784.8	760.5	761.0	Twisting of -CH_2OH
815.9	813.0	812.5	805.4	795.3	Deformation of carbon ring
873.7	867.4	866.2	867.3	875.7	Twisting of -CH_2OH
923.9	921.6	914.2	931.7	930.9	Wagging of C-H
975.9	981.0	981.8	981.3	990.1	Wagging of C-H
1089.7	1047.6	1048.2	1048.5	1057.3	Wagging of C-H and O-H
-	1097.0	1097.8	1096.9	1105.3	Wagging of C-H and O-H
-	1136.0	1123.6	1136.1	1144.5	Wagging of C-H and O-H
1265.2	1247.8	1249.4	1250.1	1258.5	Twisting of -CH_2OH
1338.5	1372.0	1358.6	1327.3	1326.7	Wagging of C-H and O-H
1400.3	1414.8	1413.6	1386.5	1387.1	Twisting of -CH_2OH
1637.5	-	-	1627.3	1617.3	Deformation of carbon ring
2025.1	-	-	-	-	-
2900.8	2978.2	2979.0	2978.9	2987.3	Stretching of C-H
3192.1	-	-	3464.5	3465.7	Stretching of O-H
3429.3	3802.6	3825.8	3779.3	3781.2	Stretching of O-H
-	3830.4	3837.2	3867.7	3865.7	Stretching of O-H

References

1. Head-Gordon, M., Pople, J. A. & Frisch, M. J. MP2 energy evaluation by direct methods. *Chem. Phys. Lett.* **153**, 503-506 (1988).
2. Becke, A. D. Density-functional thermochemistry. III. The role of exact exchange. *J. Chem. Phys.* **98**, 5648-5652 (1993).
3. Clark, S. J. *et al.* First principles methods using CASTEP. *Zeitschrift Für Kristallographie* **220**, 567-570 (2005).
4. Perdew, J. P., Burke, K. & Ernzerhof, M. Generalized Gradient Approximation Made Simple. *Phys. Rev. Lett.* **77**, 3865-3868 (1998).
5. Perdew, J. P. *et al.* Atoms, molecules, solids, and surfaces: Applications of the generalized gradient approximation for exchange and correlation. *Phys. Rev. B* **46**, 6671-6687 (1993).
6. Kleinman, L. & Bylander, D. M. Efficacious Form for Model Pseudopotentials. *Phys. Rev. Lett.* **48**, 1425 (1982).
